# Short versus conventional hydration for prevention of kidney injury during pre-TAVI computed tomography angiography

**DOI:** 10.1007/s12471-018-1133-1

**Published:** 2018-07-23

**Authors:** M. S. van Mourik, F. van Kesteren, R. N. Planken, J. Stoker, E. M. A. Wiegerinck, J. J. Piek, J. G. Tijssen, M. G. Koopman, J. P. S. Henriques, J. Baan, M. M. Vis

**Affiliations:** 1 Heart Centre, Amsterdam Cardiovascular Sciences, Amsterdam UMC, University of Amsterdam, Amsterdam, The Netherlands; 2 Department of Radiology, Amsterdam UMC, University of Amsterdam, Amsterdam, The Netherlands; 3 Department of Nephrology, Amsterdam UMC, University of Amsterdam, Amsterdam, The Netherlands

**Keywords:** Contrast media, Transcatheter aortic valve implantation, Computed tomography angiography, Acute kidney injury, Contrast-induced acute kidney injury prophylaxis

## Abstract

**Background:**

Computed tomography angiography (CTA) is required in the work-up for transcatheter aortic valve implantation (TAVI). However, CTA may cause contrast-induced acute kidney injury (CI-AKI). We hypothesised that a short (1 h, 3 ml/kg/h sodium bicarbonate) hydration protocol is not inferior to conventional (24 h, 1 ml/kg/h saline) hydration in avoiding a decline in renal function in patients with impaired renal function.

**Methods and results:**

Single-centre randomised non-inferiority trial in patients with impaired renal function who underwent pre-TAVI CTA. Patients were randomised on a 1:1 ratio to short hydration (SHORT; 1 h sodium bicarbonate, 3 ml/kg/h) or conventional hydration (CONV; 24 h saline, 1 ml/kg/h). Outcomes included percentage change in serum creatinine until 2–6 days after CTA with a non-inferiority margin of 10% and an increase on the Borg dyspnoea scale ≥1 point. Seventy-four patients were included. Increase in creatinine was 6 µmol/l (95% CI 2.5–9.3) in the SHORT versus 2 µmol/l (95% CI-1.4 to 6.3) in the CONV arm (*p* = 0.167). The percentage change was 4.6% (95% CI 2.0–7.3%) in the SHORT arm versus 2.5% (95% CI: 0.8 to 5.8%) in the CONV arm. The difference in percentage increase in creatinine between the two arms was 2.1% (95% CI: 2.0–6.2%; *p*-value non-inferiority: <0.001). CI-AKI and a need for dialysis were not observed. An increase of ≥1 point on the Borg scale (dyspnoea scale ranging from 1 (lowest) to 10 (highest)) was seen in 1 patient in the SHORT arm versus 5 patients in the CONV arm (2.9% vs 16.1%, *p* = 0.091).

**Conclusion:**

For patients with impaired renal function undergoing pre-TAVI CTA, a short 1‑h, low-volume hydration protocol with sodium bicarbonate is not inferior to conventional 24-h, high-volume saline hydration.

**Electronic supplementary material:**

The online version of this article (10.1007/s12471-018-1133-1) contains supplementary material, which is available to authorized users.

## What’s new?

- A short, 1‑h hydration with sodium bicarbonate is not inferior to conventional 24-h, high-volume sodium chloride hydration for the prevention of contrast-induced acute kidney injury (CI-AKI) in computed tomography angiography in the work-up for transcatheter aortic valve implantation.
- In the short hydration arm, a trend towards less dyspnoea was reported by patients compared to the conventional hydration strategy.
- None of the patients in the study developed CI-AKI.

## Introduction

Transcatheter aortic valve implantation (TAVI) has emerged as an appropriate treatment for patients with severe symptomatic aortic valve stenosis [1]. Prior to TAVI, contrast-enhanced computed tomography angiography (CTA) is performed for assessment of the anatomy [2, 3]. However, the iodinated contrast media used for CTA may cause contrast-induced (CI) acute kidney injury (AKI) [4]. Usually serum creatinine levels peak at 2–5 days after contrast administration. CI-AKI is associated with permanent and irreversible kidney injury and mortality rates of more than 20% [5]. Of the TAVI population, 70% have a preprocedural renal impairment and are therefore at increased risk for CI-AKI [6]. Moreover, several other factors that are often observed in TAVI patients are associated with an elevated CI-AKI risk, including advanced age, diabetes mellitus, congestive heart failure and use of nephrotoxic medication [5, 7, 8]. In order to mitigate the effects of contrast media [9–13] in patients with impaired renal function, most guidelines recommended hydration with sodium chloride 0.9% (saline) or sodium bicarbonate (1.4%) before and after contrast administration [10–12]. We hypothesised that a short hydration protocol of 1 h sodium bicarbonate is not inferior to conventional hydration with 24 h sodium chloride as regards the decline of renal function while reducing dyspnoea caused by hydration.

## Methods

### Population

The study population consisted of consecutive patients with symptomatic aortic valve stenosis and impaired renal function who underwent pre-TAVI CTA. All patients were screened for CI-AKI prevention [12]. Inclusion criteria were chronic kidney disease (CKD) classification 3a or above (estimated glomular filtration rate (eGFR) <60 ml/min/1.73 m^2^ based on the modification of diet in renal disease formula [14]), age >18 years and planned for CTA prior to TAVI. Exclusion criteria were known iodine allergy, contrast administration <7 days, already on haemodialysis, multiple myeloma, Waldenstrom’s disease and left ventricular ejection fraction <20% (not eligible to be randomised to conventional hydration).

### Study design and randomisation

The study was designed as an open-label single-centre, unblinded, randomised non-inferiority trial and followed CONSORT (Consolidated Standards for Reporting Trials) guidelines for non-inferiority trials [15]. The protocol was registered in the Netherlands Trial Register (NTR5014), complies with the Declaration of Helsinki and was approved by the local ethics committee. All participants provided written informed consent before randomisation.

Randomisation was performed electronically at admission on a 1:1 ratio to either a short hydration protocol (SHORT; 1 h sodium bicarbonate prior to CTA, 1.4% 3 ml/kg/h) or conventional hydration (CONV; 24 h sodium chloride, 0.9% 1 ml/kg/h, 8 h prior to CTA, 16 h post-CTA). Block randomisation was used (block size 4, 6 and 8), stratified into four groups corresponding to CKD classification 3a, 3b, 4 and 5. The eGFR ranges per group were (3a) 45–59, (3b) 30–44, (4) 15–29 or (5) <15 ml/min/1.73 m^2^.

### Study procedure

Prior to CTA, patients were instructed to temporarily discontinue all nephrotoxic medication on the day of CTA. According to hospital protocol all patients with impaired renal function in TAVI work-up were hospitalised on the day of CTA. Hydration volumes were based on body weight. If patients developed acute heart failure, hydration was discontinued according to hospital protocol. Contrast administration at CTA was performed with 90 ml iopromide 300 mg I/ml (Ultravist 300; Bayer, Leverkusen, Germany; low osmolar, non-ionic) at a rate of 5 ml/s. No other CI-AKI preventive measures were used. Directly prior to the start of hydration blood serum and urine samples were collected. The level of dyspnoea was scored using the Borg CR10 scale at baseline and within 1 h after completing the hydration. The follow-up blood sample was collected 2–5 days after CTA at the outpatient clinic or general practitioner and sent to the regional laboratory service for analysis.

### Outcome

The primary outcome was the percentage change in serum creatinine between the two hydration protocols at 2–5 days after contrast administration as compared to baseline creatinine. Based on previous research the non-inferiority margin for the percentage change was set at a 10% difference between the two hydration groups [16].

Predefined secondary outcomes were the absolute change in creatinine, the occurrence of CI-AKI (defined as an increase in creatinine >25% or 44.2 µmol/l within 2–5 days [17]), an increase on the self-reported level of dyspnoea according to the Borg scale and, as a marker for acute heart failure, the absolute change in *N*-terminal prohormone of brain natriuretic peptide (NT-proBNP) within 2 and 5 days. In addition we analysed the mean change in eGFR and hydration volumes.

### Statistical analysis

The trial was designed as a non-inferiority study and was powered on the relative change in creatinine based on a prior hydration trial [16]. A predefined non-inferiority margin of 10% was used. The one-sided two-sample *t*-test of equivalence of means was used to compare means. With a‑one sided α = 0.025 and β = 0.15, it was calculated that 35 patients per arm would be sufficient (70 patients in total). To correct for a dropout of approximately 15% the target sample size was determined as 80 patients.

Study results were analysed on a modified intention to treat (mITT) basis; all patients that did not receive the allocated intervention (hydration) or with failed follow-up measurements were excluded from the main analysis. Baseline categorical variables were presented as numbers with percentages, continuous variables as means with standard deviations (SD) or medians with inter-quartile ranges (IQR). Non-inferiority for the percentage change in creatinine was calculated using a two-sided, two-sample *t*-test. The mean of the difference with its 95% confidence interval was compared with the predefined non-inferiority margin; a mean difference between the two hydration protocols of less than 10% was considered non-inferior. A *p*-value for the non-inferiority test was calculated using a one-sided, two-sample *t*-test. All secondary outcomes were calculated using superiority testing applying two-sided *t*-tests or Fisher exact test as appropriate.

A *p*-value of <0.050 was considered statistically significant. All statistical analyses were performed using SPSS version 23.0 (IBM Corp, Armonk, NY, USA).

## Results

**Fig. 1:**
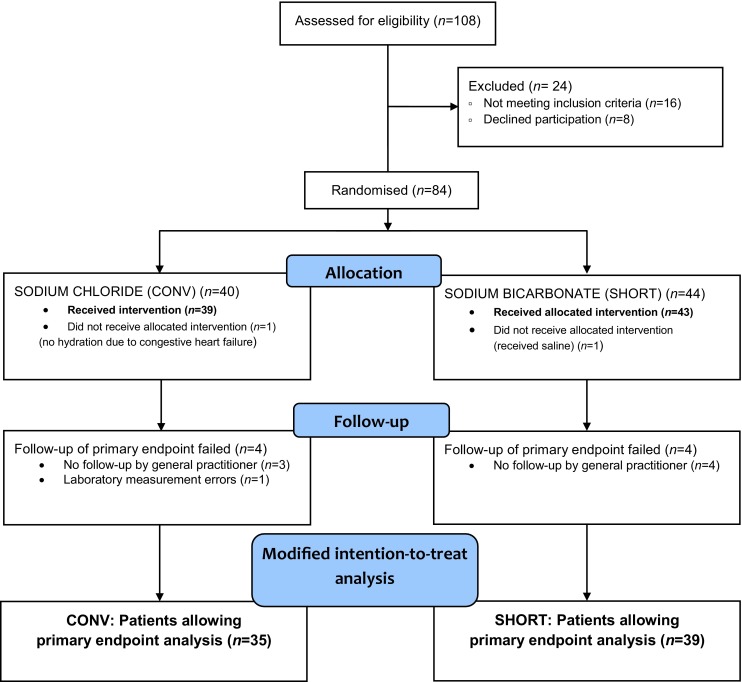
Trial profile. *CONV* conventional hydration, *SHORT* short hydration

Between January 2015 and August 2016, 84 patients were enrolled in the study and randomised to either the short or conventional hydration protocol. The primary endpoint could be analysed in 74 patients (mITT population), 39 in the SHORT arm and 35 in the CONV arm (for study profile see Fig. 1). Follow-up tests were performed after a median of 4 days in both arms (IQR 3–5 days and 3–6 days in the CONV and SHORT arm respectively). Details of the excluded patients are described in the electronic supplementary table. The median age of the study participants was 82.9 years (IQR 78.9–85.4); 41 patients were women (55.4%). The clinical characteristics of all 74 patients are described in Tab. 1. The number of patients per eGFR category was equally distributed over the two arms. No patients with eGFR <15 ml/min were enrolled (Tab. 1).

**Table 1:** Baseline demographics

	Short hydration/*Sodium bicarbonate* (*n* = 39)	Conventional hydration/*Sodium chloride* (*n* = 35)	*p*-value
Age (years)	81.2 (77.7–84.9)	83 (80.7–86.4)	0.088
Female gender—*n* (%)	19 (48.7)	22 (62.9)	0.249
BMI (kg/m^2^)	27.3 (24.1–30.1)	26.6 (23.8–28.3)	0.205
Diabetes mellitus—*n *(%)	14 (35.9)	9 (25.7)	0.452
Peripheral artery disease—*n *(%)	6 (15.4)	2 (5.7)	0.267
Hypertension—*n *(%)	32 (82.1)	26 (74.3)	0.573
Coronary artery disease—*n *(%)	22 (56.4)	13 (37.1)	0.109
COPD— *n *(%)	12 (30.8)	5 (14.3)	0.106
NYHA class III or IV—*n *(%)	29 (74.4)	24 (68.6)	0.615
LVEF <40%)—*n *(%)	9 (23.1)	4 (11.4)	0.231
AVA (cm^2^)^a^	0.90 (0.70–1.00)	0.90 (0.68–1.00)	0.994
Aortic valve maximal gradient (mm Hg)^b^	53 (38–77)	56 (44–70)	0.849
*Pre-admission eGFR*	46 (35–52)	49 (40–53)	0.168
eGFR 45–60 ml/min—*n *(%)	22 (56.4)	23 (65.7)	
eGFR 30–45 ml/min—*n *(%)	11 (28.2)	11 (31.4)	
eGFR 15–30 ml/min—*n *(%)	6 (15.4)	1 (2.9)	
eGFR <15 ml/min—*n *(%)	–	–	
Admission creatinine (µmol/l)	109 (94–135)	99 (88–119)	0.164
NT-proBNP (ng/l)	1,746 (726–3449)	1,561 (514–3354)	0.615
Microalbuminuria —*n *(%)^d^	22 (59.5)	20 (62.5)	0.810
Glycosuria—*n* (%)^e^	12 (34.3)	6 (18.2)	0.173
STS-PROM score	5,074 (3,308–6,120)	4,474 (3,092–5,540)	0.171
EuroScore I	11.69 (8.99–20.16)	11.39 (8.99–19.02)	0.782
EuroScore II	5.08 (2.78–8.24)	3.41 (2.48–5.14)	0.069
On diuretics—*n *(%)	31 (79.5)	22 (62.9)	0.129
On NSAIDs— *n *(%)	–	2 (5.7)	0.220
On other nephrotoxic medication—*n *(%)	2 (5.1)	2 (5.7)	1.00
Nephrotoxic medication stopped—*n *(%)^f^	29 (90.6)	18 (75.0)	0.278

Continuous data are presented as a median (inter-quartile range 25–75). Categorical data are presented as a number with a percentage

*BMI* body mass index, *COPD* chronic obstructive pulmonary disease, *NYHA* New York Heart Association, *LVEF* left ventricle ejection fraction, *AVA* aortic valve area, *eGFR* estimated glomerular filtration rate, *NT-proBNP N*-terminal prohormone of brain natriuretic peptide,* STS-PROM* the Society of Thoracic Surgery—predicted risk of mortality, *NSAIDs* non-steroid anti-inflammatory drugs

^a^Data missing in 11 patients in the modified intention to treat (mITT) group and in 1 of the excluded group

^b^Data missing in 6 patients in the mITT group and in 1 in the excluded group

^c^Calculated using the Modification of Diet in Renal Disease formula

^d^Determined in spot urine before hydration started and defined as an albumin/creatinine ratio of ≥3.5 mg/mmol for women and ≥2.5 mg/mmol for men; data missing in 2 patients in the short hydration group and 3 patients in the conventional hydration group

^e^Defined as any glucose in spot urine; data missing in 4 patients in the short hydration group and 2 patients in the conventional hydration group

^f^Number of patients who stopped nephrotoxic medication as a percentage of all patients that used nephrotoxic medication

**Fig. 2:**
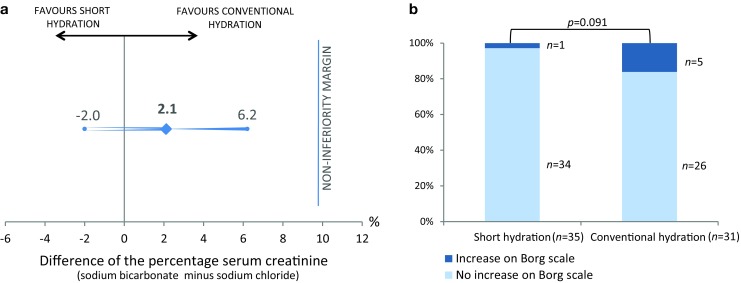
Trial outcomes. **a** Difference of serum creatinine before computed tomography angiography (CTA) and 2–5 days after CTA between the two study arms: Short hydration indicates hydration with 1 h sodium bicarbonate 1.4% (3 ml/kg/h); conventional hydration indicates hydration with 24 h sodium chloride 0.9% (1 ml/kg/h). **b** Increase of at least one point on the Borg scale per hydration group. The Borg scale of perceived exertion ranges from 0 to 10, whereby 0 = least perceived exertion, 10 = most perceived exertion

The percentage change in creatinine was an increase of 4.6% (95% CI: 2.0–7.3%) in the SHORT arm versus 2.5% (95% CI: −0.8 to 5.8%) in the CONV arm (Tab. 2). The difference in the percentage change in creatinine between the two both arms was 2.1% (95% CI: 2.0–6.2%). This was below the non-inferiority margin of 10% and corresponded to a *p*-value for non-inferiority of 0.00045 (Fig. 2a). The absolute change in serum creatinine was an increase of 6 µmol/l (95% CI: 2.5–9.3) in the SHORT arm versus an increase of 2 µmol/l (CI%: −1.4 to 6.3) in the CONV arm (*p* = 0.167). The mean eGFR change was −2.2 ml/min/1.73 m^2^ (SD: 4.3) versus 1.2 (SD: 5.8) ml/min/1.73 m^2^ in the SHORT and CONV arm respectively (*p* = 0.41; Tab. 2). None of the patients developed CI-AKI or a need for dialysis (the same applies to randomised patients excluded from the mITT analysis).

**Table 2:** Baseline and follow-up (2–5 days) differences in serum creatinine, eGFR, haemoglobin, IgG and NT-proBNP, split between short and conventional hydration

	Short hydration/*Sodium bicarbonate*			Conventional hydration/*Sodium chloride*			*p*-value of difference
	Pre	Post	Difference	Pre	Post	Difference	
Creatinine (µmol/l)^a^	118 ± 37	124 ± 42	6 ± 10	110 ± 33	112 ± 35	2 ± 11	0.167
eGFR (MDRD; ml/min/1.73 m^2^)^a^	50.7 ± 12.3	48.5 ± 12.6	−2.2 ± 4.3	52.7 (±12.5)	51.5 ± 12.7	−1.2 ± 5.8	0.409
Haemoglobin (mmol/l)^b^	7.6 ± 1.1	7.6 ± 1.1	0.1 ± 0.4	7.7 ± 1.0	7.8 ± 0.9	0.1 ± 0.5	0.900
IgG (g/l)^c^	10.3 ± 3.6	10.5 ± 3.8	0.2 ± 0.9	10.0 ± 2.9	10.6 ± 3.1	0.6 ± 1.0	0.171
NT-proBNP (ng/l)^d^	1,630 (642–3,815)	1,174 (209–2,024)	−100 (−1,449 to 176)	1,326 (352–3,294)	755 (288–1,704)	75 (−886 to 371)	0.342

*p*-values represent the difference between randomisation groups in the change in blood markers

*eGFR* estimated glomerular filtration rate,* MDRD* modification of diet in renal disease, *IgG* immunoglobulin G, *NT-proBNP N*-terminal brain natriuretic peptide. The numbers of patients included in the analysis, sodium bicarbonate and sodium chloride respectively: ^a^*n*: 39–35, ^b^*n* : 37–33, ^c^ *n* : 27–30, ^d^ *n* : 25–24

The median hydration volume was 228 ml (IQR: 204–261) for patients in the SHORT arm versus 1,759 ml (IQR: 1,536–1,920) in the CONV arm. One patient randomised to the CONV protocol developed acute decompensation at the end of hydration for which the fluid administration was stopped and intravenous furosemide (40 mg) given once.

Data completion of the Borg scale was successful in 66 patients (35 patients in the SHORT and 31 in the CONV arm). An increase in the level of dyspnoea by at least one point on the Borg scale was seen in 1 patient in the SHORT arm (2.9%) compared to 5 patients in the CONV arm (16.1%; *p* = 0.091; Fig. 2b). There was no difference in left ventricular function between patients with an increase in level of dyspnoea compared to those without (*p* = 0.580). There was no change in the level of NT-proBNP measured between 2 and 5 days post-CTA in the two arms; the difference in NT-proBNP before and after hydration was −100 ng/l (IQR: −1,449 to 176) in the SHORT versus 75 ng/l (IQR: −866 to 371) in the CONV arm (*p* = 0.342; Tab. 2).

## Discussion

In this randomised non-inferiority trial we are the first to demonstrate that for pre-TAVI CTA, a short hydration protocol with 1 h sodium bicarbonate (3 ml/kg/h) is not inferior to conventional hydration with 24 h sodium chloride (1 ml/kg/h) and does not result in a significant decline in renal function. The difference in the change of creatinine was below the non-inferiority margin. The absolute changes in creatinine were small (only 6 µmol/l and 2 µmol/l for short and conventional hydration respectively, which is well below the clinically significant change in creatinine.

Our results are in line with a randomised clinical trial in a mixed population of 570 patients undergoing CTA in which 1 h hydration with 250 ml sodium bicarbonate (1.4%) prior to scanning was described to be as effective as 2,000 ml saline (1,000 ml prior to and 1,000 ml after CTA) to prevent decline of renal function [16]. A newer study even suggests that no hydration prophylaxis is necessary at all in patients with an eGFR ≥30 ml/min/1.73 m^2^ [18]. In this study we observed a trend towards more hydration-induced dyspnoea in the patients hydrated according to the conventional procotol.

### Acute heart failure

Hydration with the short protocol has the advantage of lower fluid load administration, decreasing the risk of acute heart failure. In our trial, only one patient hydrated with the conventional protocol developed acute heart failure. We used NT-proBNP as a marker for decompensation and found no difference between the two hydration arms at 2–5 days after contrast administration. Shortness of breath was a more sensitive symptom of developing acute heart failure in this population, increasing the importance of dyspnoea assessment. Although the study sample was not powered to detect any statistical differences on the Borg CR10 dyspnoea scale, we did see a substantial difference in patients with increased dyspnoea between the two groups.

### Study limitations

This single-centre study had its limitations. We had a small but appropriate sample size for the primary endpoint. Although there were no patients in our study who developed CI-AKI, we are aware that the definition of CI-AKI is based on the cut-off values for serum creatinine change and differs across guidelines [19–21]. Our primary outcome was the percentage change in serum creatinine. We used a common definition for the development of CI-AKI, which is not the most strict definition but is predictive for long-term mortality and corresponds closely to a high degree of kidney injury according to the RIFLE criteria (risk, injury, failure, loss of kidney function, and end-stage kidney disease ) and the Acute Kidney Injury Network (AKIN) [19–21]. Even when analysing our data using the most strict definitions to detect kidney injury, none of the patients were considered to have developed CI-AKI. This is remarkable, since most studies with follow-up after CTA report at least a small percentage of patients developing CI-AKI. A possible reason could be that despite the broad inclusion criteria, most patients in our study had a relatively ‘high’ eGFR >30 ml/min/1.73 m^2^, with a lower risk of developing CI-AKI. Nevertheless, this distribution of eGFR represents our current TAVI cohort and the results can be interpreted as those of a real-world TAVI population. Furthermore, this is a population at high risk of developing renal injury based on their high age and a high prevalence of diabetes mellitus, hypertension and albuminuria. This would make it easier to extend the results to other patient groups at lower risk for CI-AKI.

Lastly, although we examined dyspnoea, this study was underpowered to detect a significant difference in patient-perceived exertion. The trend towards more dyspnoea in patients hydrated with the conventional protocol was clear but not significant.

### Future directions and implementation of the results

We included a homogeneous but complex population of high age, with severe aortic valve stenosis and multiple comorbidities, prone to develop CI-AKI. Since the short hydration protocol was feasible and safe for these patients, a more general population is likely to benefit equally from the same strategy.

New indications for CTA with similar amounts of contrast, such as coronary CTA, are emerging. Most likely implementation of a short hydration protocol will induce more flexible CTA planning in an outpatient setting, a reduction of admission days as well as of the risk of hospital-acquired complications, and a reduction of costs while improving patient comfort [16].With the current technological advances in CTA, the volume of contrast agent necessary per study for quality imaging is decreasing [22, 23]. It is conceivable that with current techniques, the risks for CI-AKI after CTA will diminish over time.

Our findings are in line with recent guideline changes by the Dutch Society for Radiology and embedded in the guideline database of the Dutch Society for Clinical Specialists [24].

## Conclusion

For patients undergoing pre-TAVI CTA, short and low-volume 1 h hydration with sodium bicarbonate is not inferior to conventional, 24-h high-volume sodium chloride hydration as CI-AKI prophylaxis. The non-inferiority warrants safe clinical implementation of this short hydration protocol in daily clinical practice for pre-TAVI CTA. Since patients with severe CKD were underreported in this study, the results might be limited to patients with an eGFR > 30 ml/min/1.73 m^2^.

## Caption Electronic Supplementary Material

Supplemental Table 1: Demographic and clinical characteristics of all patients who underwent randomization. Modified intention to treat (mITT)-population versus the randomized but excluded population
