# Supplementary material for: Short versus conventional hydration for prevention of kidney injury during pre-TAVI computed tomography angiography
Source: Neth Heart J. 2018 Jul 23;26(9):425–32. doi: 10.1007/s12471-018-1133-1 (PMC6115307; doi:10.1007/s12471-018-1133-1)
Supplement: Supplementary file 1 — Supplemental Table 1: Demographic and clinical characteristics of all patients who underwent randomization. Modified intention to treat (mITT)-population versus the randomized but excluded population [file 12471_2018_1133_MOESM1_ESM.doc]

Supplemental Table 1: Demographic and clinical characteristics of all patients who underwent randomization

Modified intention to treat (mITT)-population versus the randomized but excluded population

| ***Age– years (IQR)*** |  | **82.9 (78.9-85.4))** | **84.9 (81.2-87.2)** |  | **0.11** |
| --- | --- | --- | --- | --- | --- |
| ***Female gender – n (%)*** |  | 41 (55.4) | 5 (50.0) |  | 0.75 |
| ***BMI - kg/m^2^ (IQR)*** |  | 26.8 (23.8-29.7) | 28.4 (23.1-33.6) |  | 0.48 |
| ***Diabetes mellitus – n (%)*** |  | 23 (31.1) | 6 (60.0) |  | 0.087 |
| ***Peripheral artery disease – n (%)*** |  | 8 (10.8) | - |  | 0.59 |
| ***Hypertension – n (%)*** |  | 58 (78.4) | 6 (60.0) |  | 0.24 |
| ***Coronary artery disease– n (%)*** |  | 35 (47.3) | 3 (30.0) |  | 0.50 |
| ***COPD – no (%)*** |  | 17 (23.0) | 2 (20.0) |  | 1.00 |
| ***NYHA class III or IV– n (%)*** |  | 53 (71.6) | 6 (60.0) |  | 0.48 |
| ***LVEF < 40% – no (%)*** |  | 13 (17.6) | 2 (20.0) |  | 1.00 |
| ***AVA cm2 ^*^*** |  | 0.90 (0.70-1.00) | 0.69 (0.56-0.85) |  | 0.053 |
| ***Aortic valve maximal gradient* †** |  | 54 (42.71) | 60 (47-85) |  | 0.267 |
| ***SPAP> 60 mmHg – n (%)*** |  | 2 (2.7%) | 1 (10%) |  | 0.320 |
| ***Pre-admission eGFR ml/min ‡*** |  |  |  |  | 0.81 |
| ***eGFR 45-60 ml/min – n (%)*** |  | 45 (60.8) | 6 (60.0) |  |  |
| ***eGFR 30-45 ml/min – n (%)*** |  | 22 (29.7) | 2 (20.0) |  |  |
| ***eGFR 15-30 ml/min – n (%)*** |  | 7 (9.5) | 2 (20.0) |  |  |
| ***eGFR <15 ml/min – n (%)*** |  | - | - |  |  |
| ***Admission creatinine – µmol/L (IQR)* *§*** |  | 106 (91-129) | 118 (102-186) |  | 0.069 |
| ***NTproBNP – ng/L (IQR) §*** |  | 1681 (549-3406) | 2103 (716-2860) |  | 0.82 |
| ***Microalbuminuria∥ – n (%)*** |  | 42 (60.9) | 4 (50.0) |  | 0.71 |
| ***Glycosuria*#*– n (%)*** |  | 18 (26.5) | 3 (37.5) |  | 0.68 |
| ***STS-PROM – score (IQR)*** |  | 4.656 (3.112-5.863) | 5.778 (3.422-10.871) |  | 0.25 |
| ***Logistic EuroScore I – score (IQR)*** |  | 11.66 (8.99-19.51) | 11.62 (7.98-19.51) |  | 0.68 |
| ***EuroScore II – score (IQR)*** |  | 4.24 (2.63-7.44) | 3.91 (2.78-8.72) |  | 0.85 |
| ***Nephrotoxic Medication use –n (%)*** |  | 56 (75.7) | 8 (80.0) |  | 1.00 |
| ***-Diuretics – n (%*** |  | 53 (71.6) | 8 (80.0) |  | 0.72 |
| ***-NSAIDs – n (%)*** |  | 2 (2.7) | - |  | 1.00 |
| ***-Other nephrotoxic medication – n (%)*** |  | 4 (5.4) | - |  | 1.00 |
| ***Nephrotoxic medication stopped**– n (%)*** |  | 47 (83.9) | 4 (50.0) |  | 0.047 |

Patients were excluded from the modified intention to treat population if the patient did not receive the allocated hydration protocol (n=2) or if the creatinine measurement after hydration failed (n=8)

BMI denotes body mass index; COPD chronic obstructive pulmonary disease; NYHA New York Heart Association; LVEF left ventricle ejection fraction; SPAP systolic pulmonary artery pressure; eGFR estimated Glomerular Filtration rate; STS-PROM the Society of Thoracic Surgery – predicted risk of mortality; NSAIDs non-steroid anti-inflammatory drugs.

AVA Aortic Valve Area in cm^2^***; ^*^*** data was missing in 11 patients in the MITT group and in 1 of the excluded group; **†**Aortic valve maximal gradient, data was missing in 6 patients in the MITT group and in 1 of the excluded group

***‡*** eGFR was calculated using the Modified of Diet in Renal Disease (MDRD) formula; *§* data was missing in 2 patients in the excluded group; ∥Microalbuminuria was determined in spot urine before hydration started and defined as an albumin/creatinine ratio of ≥3.5 mg/mmol for women and ≥2.5 mg/mmol for men, data was missing in 5 patients in the MITT group and 2 patients in the excluded group; # Glycosuria was defined as any glucose in spot urine, data was missing in 6 patients in de MITT group and 2 patients in the excluded group; **Number of patients stopped with nephrotoxic medication as a percentage of all patients that used nephrotoxic medication.
